# Unexpected disseminated histoplasmosis detected by bone marrow biopsy in a solid organ transplant patient

**DOI:** 10.1002/ccr3.1282

**Published:** 2017-11-22

**Authors:** Caroline T. Simon, Carlos A. Murga‐Zamalloa, Michael A. Bachman, Lindsay A. Petty, Sarah M. Choi

**Affiliations:** ^1^ Department of Pathology University of Michigan Ann Arbor Michigan USA; ^2^ Department of Internal Medicine University of Michigan Ann Arbor Michigan USA

**Keywords:** Bone marrow, ferritin, hemophagocytic lymphohistiocytosis, histoplasmosis

## Abstract

Disseminated histoplasmosis and hemophagocytic lymphohistiocytosis show overlapping features, which require careful contextual interpretation. Histopathologic evaluation can potentially rapidly identify cases of possible histoplasmosis. A high index of clinical suspicion, particularly in endemic areas and in a setting of immunosuppression, is critical to appropriate diagnosis and treatment.

A 29‐year‐old man with a history of end‐stage renal disease secondary to minimal change disease, status postrenal transplant 2 years prior, presented with a one‐week history of fever, night sweats, chills, generalized aches, and diarrhea. His immunosuppressive regimen included tacrolimus, cyclosporine, and prednisone. The patient lives in Michigan and works as a truck driver with most recent travel to Arizona and Texas. Following admission, he was noted to be tachycardic and febrile (up to 40.1°C), but his physical examination was otherwise unremarkable. CBC showed mild anemia (Hgb as low as 11.1 g/dL) and variable thrombocytopenia (platelets as low as 115 k/μL). Imaging studies were significant for ill‐defined centrilobular groundglass opacities in the lungs, although without any respiratory complaints or hypoxia initially. PET/CT also noted hypermetabolic lymph nodes, spleen (without splenomegaly), and a soft tissue mass in the porta hepatis, raising the possibility of a post‐transplant lymphoproliferative disorder; however, a biopsy was not performed during the admission based on the subsequent findings.

He was initially placed on empiric therapy with vancomycin and piperacillin–tazobactam and over the next 5 days underwent an extensive workup including blood and urine cultures; CMV, HIV, EBV, HHV6, parvovirus, toxoplasma, adenovirus, and HSV PCR; Q fever, *Bartonella*,* Brucella*, parvovirus and fungal serology; and *Clostridium difficile* antigen and toxin and serum cryptococcal and urine *Legionella* antigen. These results were all negative. Concurrently, he was found to have hyperferritinemia of 14,914 ng/mL (normal range 18–320 ng/mL) and triglycerides 228 mg/dL, concerning for hemophagocytic lymphohistiocytosis (HLH). To evaluate for hemophagocytosis, soluble CD25 was measured at 2291 U/mL, fibrinogen was 415 mg/dL, and a bone marrow biopsy was performed; NK‐cell activity was not measured. The core biopsy showed normocellular bone marrow with trilineage hematopoiesis.

A single histiocyte with oval‐shaped organisms was seen on the aspirate smear (Fig. 1A). The morphological differential diagnosis included *Histoplasma capsulatum*,* Leishmania* species, *Candida glabrata, Blastomyces dermatitidis*, among others 1. Grocott‐methenamine silver stain performed on the bone marrow core demonstrated additional histiocytes containing multiple yeast forms exhibiting narrow‐based budding (Fig. 1B). Rare hemophagocytes were noted (Fig. 1C). Based on these findings, intravenous liposomal amphotericin was started. Subsequent urine *Histoplasma* antigen test results (above the limit of quantification) and fungal blood culture results returned, confirming a diagnosis of *H. capsulatum*.

**Figure 1:**
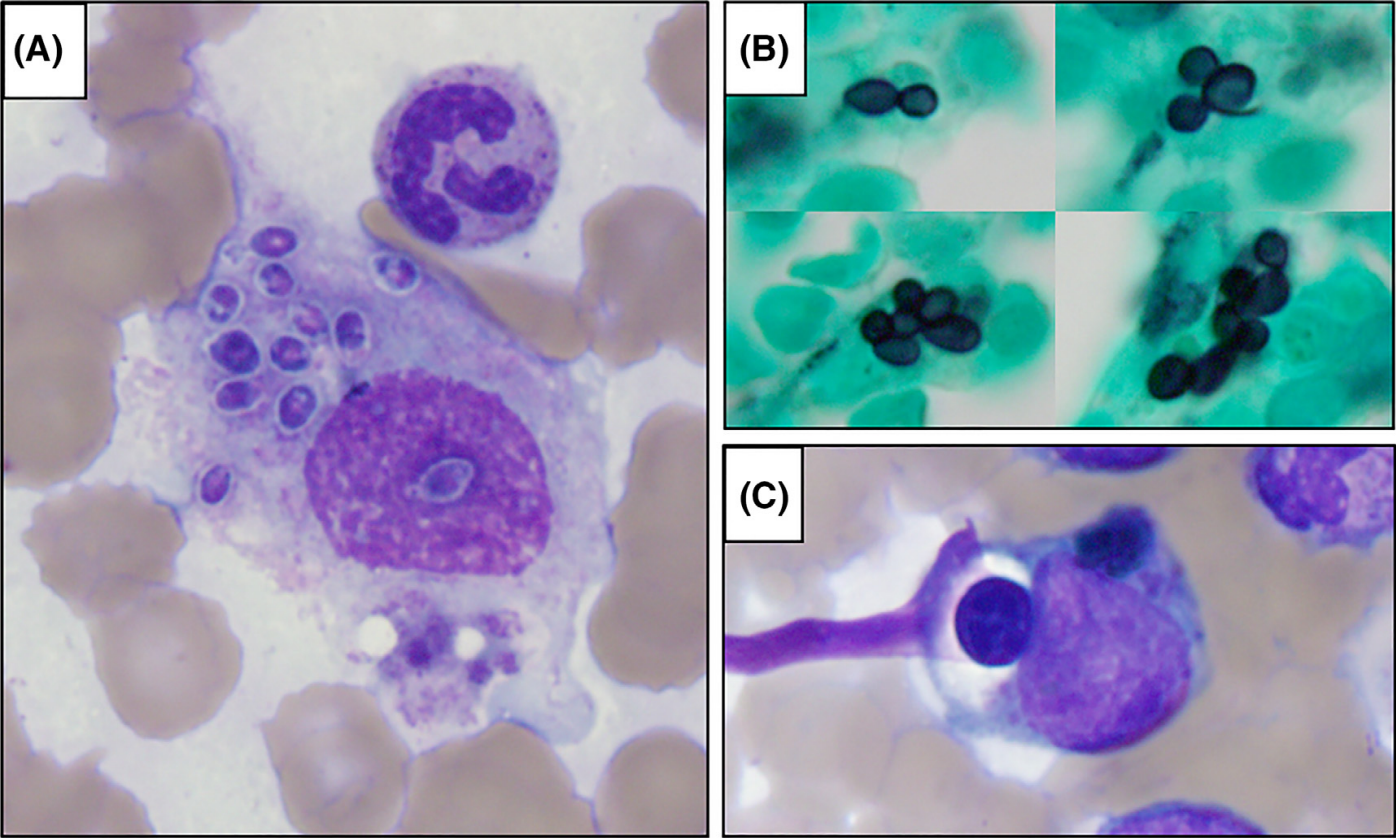
A. Wright‐Giemsa stained bone marrow aspirate showing multiple intracellular organisms within a histiocyte. B. Grocott methenamine stain highlights additional organisms on the core biopsy. C. Example of hemophagocytosis in bone marrow aspirate.

Disseminated histoplasmosis is predominantly seen in immunosuppressed patients, including solid organ transplant patients 2. An association between hemophagocytic lymphohistiocytosis (HLH) and *H. capsulatum* infection has been observed, commonly in the setting of AIDS 3, 4. The diagnostic criteria for HLH in the 2004 guidelines include fever, splenomegaly, cytopenias affecting at least two of the three lineages, hypertriglyceridemia and/or hypofibrinogenema, hemaphagocytosis in the bone marrow spleen or lymph nodes, low/absent NK‐cell activity, hyperferritinemia and high levels of sIL‐2r 5. The patient met three of these (fever, elevated ferritin, and rare hemophagocytosis), which was insufficient for diagnosis of HLH, although soluble CD25, triglycerides and cytopenias did approach meeting criteria. HLH can be seen either as a primary hereditary disorder or secondary to a number of causes including infection (particularly viral, e.g., EBV), malignancy, and rheumatologic disease. It is important to note that hyperferritinemia alone is not specific for HLH, as this finding in isolation can be seen in the setting of inflammation and infection, even with indolent course 6. *Histoplasma* infection in particular can cause elevated ferritin and fever without meeting other criteria for HLH. It is also important to point out that hemophagocytosis seen on bone marrow aspirate is not specific to HLH 7, 8. In a study by Rivière et al. 8, hemophagocytosis was found in 39.4% of patients where the diagnosis of HLH was suspected but ultimately ruled out, as in this case.

This case demonstrates an instance where the bone marrow biopsy was particularly useful in early detection of the patient's infection, as some of the confirmatory assays require more extended periods of time to result. Although bronchoalveolar lavage (BAL) and respiratory cultures were not performed in the current case, histopathologic evaluation of these samples could also potentially have yielded similarly analogous information. A high index of clinical suspicion for infection in potential patients with HLH who are immunosuppressed is necessary, both in the clinical and pathologic practice, especially in endemic areas. This patient was continued on intravenous liposomal amphotericin followed by voriconazole. He exhibited complete response with immediate reduction in fever and resolution of all imaging abnormalities and symptoms.

In subsequent monitoring, the ferritin initially increased to >16,500 ng/mL in the 2 days prior to bone marrow biopsy and decreased to 3602 ng/mL 5 days after the bone marrow biopsy was performed with no additional follow‐up.

## Authorship

CTS: wrote the manuscript and created the Figure. CAM‐Z: wrote the manuscript. MAB: provided additional comments, direction, and editing. LAP: provided additional comments, direction, and editing. SMC: wrote the manuscript.

## Conflict of Interest

None declared.
